# Challenges in replication: Does amygdala gray matter volume relate to social network size?

**DOI:** 10.3758/s13415-024-01185-w

**Published:** 2024-03-28

**Authors:** Rankin W. McGugin, Alexandra Roche, Jonathan Ma, Isabel Gauthier

**Affiliations:** Department of Psychology, Vanderbilt University, 111 21st Avenue South, Nashville, TN 37240 USA

**Keywords:** Amygdala volume, Replication, Social network size, Brain imaging

## Abstract

In this work, we tried to replicate and extend prior research on the relationship between social network size and the volume of the amygdala. We focused on the earliest evidence for this relationship (Bickart et al., *Nature Neuroscience 14*(2), 163–164, 2011) and another methodologically unique study that often is cited as a replication (Kanai et al., *Proceedings of the Royal Society B: Biological Sciences, 279*(1732), 1327–1334, 2012). Despite their tight link in the literature, we argue that Kanai et al. (*Proceedings of the Royal Society B: Biological Sciences, 279*(1732), 1327–1334, 2012) is not a replication of Bickart et al. *Nature Neuroscience 14*(2), 163–164 (2011), because it uses different morphometric measurements. We collected data from 128 participants on a 7-Tesla MRI and examined variations in gray matter volume (GMV) in the amygdala and its nuclei. We found inconclusive support for a correlation between measures of real-world social network and amygdala GMV, with small effect sizes and only anecdotal evidence for a positive relationship. We found support for the absence of a correlation between measures of online social network and amygdala GMV. We discuss different challenges faced in replication attempts for small effects, as initially reported in these two studies, and suggest that the results would be most helpful in the context of estimation and future meta-analytical efforts. Our findings underscore the value of a narrow approach in replication of brain-behavior relationships, one that is focused enough to investigate the specifics of what is measured. This approach can provide a complementary perspective to the more popular “thematic” alternative, in which conclusions are often broader but where conclusions may become disconnected from the evidence.

**Supplementary Information:**

The online version contains supplementary material available at 10.3758/s13415-024-01185-w.

## Introduction

Our study seeks to replicate claims about the relationship between social network size and grey matter volume of the amygdala—a subcortical brain region known for its involvement in emotion, cognition, and social behavior (Pessoa, 2010). Humans, like other primates, possess a large brain-to-body ratio. One proposal is that this is at least in part driven by the complexity of their social interactions (Dunbar, 1993; 1998). This theory has been challenged, however, with other work suggesting diet quality may be as or more important in determining brain size across species (DeCasien & Higham, 2019). Setting aside interspecies variability, others have suggested that individual differences in social group size in humans should be linked to variations in the brain regions that are relevant to social cognition. We address a specific offshoot of this larger claim: relating amygdala volume with various social network metrics. A landmark study by Bickart et al. (2011) (hereafter BI2011) reported that the size and complexity of real-world social networks predict overall amygdala volume, bilaterally. We revisit the evidence for the correlation through a replication of this specific analysis linking amygdala volume to the size of social networks. We also examine evidence for a variation of this claim, based on another study by Kanai et al. (2012a, 2012b) (hereafter KA2012) that was published soon after BI2011 and is often co-cited. The authors of these articles did not propose a specific mechanism for these effects. KA2012 explicitly noted that those with a larger amygdala could be better equipped to build large social networks, but that it also was possible that the experience of maintaining a larger social network could influence amygdala size. In the following paragraphs, we highlight challenges present in the existing literature, which make it difficult to get a clear picture of whether the main result from BI2011 has been supported in later work.

Readers reviewing the literature on the brain correlates of social cognition may be struck by the variability in the measurement methods of both social network size and amygdala structure. Important differences between brain morphometry approaches (Goto et al., 2022a, 2022b) are often ignored and, as a result, entirely different dependent variables can be mistaken as equivalent. Authors often refer to KA2012 as a replication of BI2011, even though neither the brain measures nor the measures of social network were the same. There is increasing concern that many reported correlations between brain structure and behavior do not replicate and that very large samples (some argue in the thousands, although see Libedinsky et al., 2022) may be required to achieve sufficient power (Genon et al., 2022; Marek et al., 2022). Such sample sizes often are not feasible, although a meta-analytic approach offers promise (Libedinsky et al., 2022; Matsumoto et al., 2023). It is in this spirit that we conducted this replication study. As we planned a brain structure-focused project relating visual object and face recognition abilities to cortical thickness (McGugin & Gauthier, in preparation), we used the opportunity to reexamine BI2011’s central claim of the relationship between the volume of the amygdala and the size of a person’s social network. In a study trying to replicate 17 different structural brain-to-behavior associations (Boekel et al., 2015), a correlation between real-world social network size and the volume of the right amygdala was the only effect that was successfully replicated. However, both positive replications (Von Der Heide et al., 2014) and failures to replicate (Lewis et al., 2011) have been reported.

### Differences in measures of social network

We focus on two early studies, BI2011 and KA2012, because they often are cited together, but there are important differences between them that should be highlighted. BI2011 measured the size and complexity of their participants’ real-world social network. KA2012 initially focused on a measure of online social network, but in a follow-up study, collected data on the size of the real-world network in a subset of their participants. As a result, together the two studies use four different measures of social network size that vary in their focus on real-world (both BI2011 and KA2012) or online (only KA2012) contacts, including the number of individuals or the structure of the social network. On the one hand, these variables are strongly related theoretically (and to some extent empirically). On the other hand, some of the analyses report partial correlations with one of these measures when controlling for the other, indicating they are considered partially distinct.

### Differences in measures of neural correlates

Even when considering only studies limited to the structural measurement of the amygdala, different measures have been used to index the size of this structure. This is in part because the options available for brain morphometry, and the way they are described, have changed over time (Goto et al., 2022a, 2022b). From a standard structural brain scan, it is possible to obtain several measures of brain structure using an approach termed surface-based morphometry (SBM; Dale et al., 1999). SBM can provide experimenters with both cortical thickness (CT, measured in 2D units) and gray matter volume (GMV, measured in 3D units). The analyses in BI2011 measured GMV. KA2012 used a different method called voxel-based morphometry (VBM; Ashburner & Friston, 2000). Voxel-based morphometry has been criticized as a cruder approach than SBM for the measurement of brain anatomy (Boekel et al., 2015). Voxel-based morphometry relies on a soft segmentation approach; instead of labeling each voxel as “gray,” “white,” or “CSF,” it returns the gray matter density (GMD, unitless), a ratio of gray matter for each voxel. Many VBM studies use an optional scaling factor.^1^ These differences result in unique names for similar measures, but also sometimes mask potentially important differences that can make some measures of size incommensurate. For instance, KA2012 discusses their dependent variable as either GMD or GMV, while others have called it “modulated” GMD. Modulated maps of GMD in fact are the multiplication of GMD and GMV at each voxel. To make matters more complicated, modulated GMD is sometimes called grey matter mass (GMM; Gennatas et al., 2017a, 2017b; Herculano-Houzel et al., 2008). These different ways to measure local brain size should not be assumed to be equivalent or even strongly correlated. For instance, GMV and CT tend to diminish as individuals age (and GMM to a lesser extent), whereas GMD experiences a pronounced increase in relation to age. Gender effects in GMV and GMD can run in opposite directions (Gennatas et al., 2017a, 2017b). In the present work, we used SBM to estimate GMV for three reasons. First, this is the measure used in BI2011, which we aimed to replicate. Second, SBM is more often reported as superior to VBM (Goto et al., 2022a, 2022b). Third, we found that the relationship between online social network and the amygdala by KA2012 often is cited as if it was based on GMV (so it is important to ask how such a relationship would replicate).

In the present work, we replicated the methods of BI2011, as well as the measures of social network size used in KA2012, with some adaptations. Our confirmatory analyses focus on the relationship between social network size and GMV in the right and left amygdala. This includes examining how real-world social networks correlate with GMV when controlling for online social network size (and vice versa). In addition, we conduct exploratory analyses that decompose the amygdala into separate components, focusing on two subdivisions in particular: the basolateral amygdala and the central amygdala. In recent years, methods that were not available to BI2011 and KA2012 were developed for the automated measurement of more than a dozen different nuclei within the amygdala. These nuclei are richly interconnected but structurally and functionally distinct (LeDoux, 2007; Sah et al., 2003). The basal, lateral, and accessory basal nuclei share similar cell types, neurotransmitters, and connectivity and, together, form the basolateral nucleus complex (BLA), which serves as the principal input region and integrates somatosensory, visual, auditory, and visceral inputs to generate social behavior (deCampo & Fudge, 2012). The central nucleus (Ce), which exhibits distinct connectivity patterns compared with the BLA, serves as the primary output nucleus of the amygdala (Janak & Tye, 2015; LeDoux, 2007). Previous work has linked the BLA and the Ce with social behavior (Jones et al., 2020a, 2020b; Wellman et al., 2016). Results for the remaining nuclei of the amygdala, including the medial, paralaminar, corticoamygdaloid transition area, and the anterior amygdaloid area will be included in the supplemental section.

## Method

### Participants

We collected data from 128 subjects from the Vanderbilt community and surrounding communities. All participants received monetary compensation for their participation. The participants were recruited for a study focusing on the correlates between laminar cortical thickness and visual abilities. Initially, we wanted to scan 150 participants, but data collection was limited because of time and funding resources spread thin because of the COVID pandemic. Each participant reported normal or corrected-to-normal vision and gave written informed consent in accordance with guidelines of the Vanderbilt University Institutional Review Board and Vanderbilt University Medical Center. Two subjects were excluded due to excessive head motion in the MRI portion of the experiment. Another nine subjects were excluded due to incomplete social network survey data. Our final sample for this study consisted of 117 subjects (71 identifying as women and the rest as men, age *M* = 22.0, *SD* = 5.8, range = 18–63 years). Most of these subjects reported being right-handed (n = 113), whereas two reported being left-handed and another two reported being ambidextrous.

### Measures of social network size

To estimate the size and complexity of participants’ social networks, online and in the real-world, we use four separate measures (SNI-Size, SNI-Complexity, SNS-Online, SNS-RealWorld), explained below and derived from two different surveys, as included in BI2011 and KA2012.

The first survey was the Social Network Index (SNI, Cohen et al., 1997) as used in by BI2011. The SNI consists of a 12-item questionnaire, and it produces two subscales. The *Number of People in Social Network Subscale* (SNI-size) estimates the size of social networks by aggregating the people with whom an individual interacts with at least once every 2 weeks. The *Number of Embedded Networks Subscale* (SNI-complexity) estimates the complexity of social networks by aggregating different groups of at least four members with whom an individual talks at least once every 2 weeks. Because self-report of the number of people in one’s social network (SNI-size) is skewed, we applied a square root transformation.

The second survey was adapted from a questionnaire adapted from Stileman (2007a, 2007b) and was used in KA2012 to measure the size of online and real-world social networks. The questionnaire consisted of 11 questions. The questions used here were identical to those reported in (Stileman, 2007a, 2007b) with one exception. The original question about Facebook (“How many friends do you have on Facebook”) was replaced, because Facebook was much less dominant during data collection in years 2021–2022 among college students than in 2012. The following three questions were used in its place: Q9. “*How many different social media platforms (e.g., Facebook, Twitter, Instagram, etc.) do you use on a regular basis (at least once a week)?*” Q10. “*How many friends or mutual followers (people you follow who ALSO follow you) do you have across all platforms? Give your best estimate*.” Q11. “*Some individuals may be friends or mutual followers with you on several platforms. How many UNIQUE friends or mutual followers do you have total? A person only counts once, even if you encounter them on many platforms. Give your best estimate*.” Question 9 was included (but not used in calculations) to prime individuals to think about all the platforms they used before estimating the number of friends and followers. Questions 10 and 11 were included in case there could be a large difference between them, but they were so highly correlated (r = .89) that we decided to only use Q11. Therefore, following KA2012, we derived two measures based on this questionnaire: SNS-Online (Q11, with square root applied to reduce skewness) and SNS-Real-World (Q1-Q8 inclusively, with square root applied, z-scored and averaged). See supplemental section for the full questionnaire.

### MRI acquisition

All participants were imaged using a whole body 7T MRI scanner in combination with a quadrature, head only transmit coil and a 32-channel receive coil array. In each participant, imaging was separated into three stages, consisting of whole brain anatomic imaging, functional localization, and ultra-high resolution susceptibility weighted imaging. Neither functional images nor ultra-high resolution susceptibility weighted images were used in this study. With our 0.7-mm isotropic voxels at 7-Tesla, our magnetic field strength and spatial resolution are higher than that in BI2011. Our data were down sampled to 1-mm isotropic resolution before segmentation in FreeSurfer (FS). Although BI2011 do not report what version of FS they used, based on publication date, it is logical to assume their data were analyzed with v5.0.0 or earlier. Our data were processed with v7.4.0, after nearly a dozen years of research-based improvements to the automatic segmentation pipeline (Goto et al., 2022a, 2022b).

Sagittal whole-brain T1-weighted images were collected with a 3D MPRAGE sequence (TR = 4.8 ms; TE = 2.1 ms; TFE inversion delay = 1300 ms; TFE shot interval = 4500 ms; flip angle = 7°; FOV = 246 x 246 x 174.3; matrix = 352 x 352 x 249; resolution = 0.7-mm isotropic).

### MRI analyses

Cortical reconstruction and segmentation of our whole-brain anatomical images was performed using FreeSurfer (FS) v7.4.0 image analysis suite (Dale et al., 1999; Fischl & Dale, 2000), with amygdala segmentation (Saygin et al., 2017). For each subject, research assistants in our lab inspected the automated segmentation results for manual corrections. No adjustments were needed. The automated FreeSurfer processing pipeline included motion correction, nonuniform intensity normalization for intensity inhomogeneity correction, removal of nonbrain tissue, transformation to Talairach space, and segmentation of the subcortical white matter and deep gray matter volumetric structures (Dale et al., 1999; Fischl et al., 2002). We selected FreeSurfer because of its reported high reproducibility in subcortical segmentations compared with other automated methods (Velasco-Annis et al., 2018)*.*

Segmentation of the amygdala and amygdala subregion volumes is based on combining manual labels from ex vivo and in vivo T1 scans to generate an atlas of amygdala nuclei (Saygin, 2017). FreeSurfer’s automated Bayesian segmentation technique establishes nine distinct amygdala nuclei per hemisphere: the lateral nucleus, basal nucleus, accessory basal nucleus, anterior amygdaloid area (AAA), central nucleus (Ce), medial nucleus, cortical nucleus, cortical amygdaloid transition area, and para-laminar nucleus. Due to functional and structural similarity, we summed the basal, lateral, and accessory basal nuclei to represent the BLA. The Ce is considered separately as the main hub for signals coming out of the amygdala (Janak & Tye, 2015; LeDoux, 2007).

Thus, our analyses focused on four volumetric measures bilaterally: total amygdala (reported in BI2011 and KA2012), total hippocampus (used as a control region in BI2011), and the BLA and Ce subregions of the amygdala, as the main input and output pathways in the amygdala respectively. Estimated total intracranial volume (eTIV) was also measured and was controlled (with age and gender) in all our analyses.

### Bayesian analysis

We used JASP (JASP Team, 2023) to conduct Bayesian tests comparing the support for different hypotheses. When we had expectations for a positive correlation, we estimated the support for a positive correlation relative to one that was null or 0 (BF+0). We sometimes also calculated the BF01 as a follow-up test to estimate the support for a null correlation versus any correlation. Priors are reported in figure legends and following best practices, we considered the robustness of the results to a variety of priors. Following Jeffreys (1961) and others, we use the following criteria for interpretation of BF values, with values between 1 and 3 being not worth more than a mention, values between 3 and 10 representing substantial evidence, values between 10 and 30 representing strong evidence, values between 30 and 100 representing very strong evidence, and those above 100, decisive evidence.

## Results

### Social network measures

Descriptive statistics for the social network measures are reported in Table 1. Two extreme values (14.90 and 17.89) for SNI-Size were winsorized to the next highest value (10.86). Before winsorization, skewness for this variable was 1.15 and kurtosis was 8.51. Table 2 reports the pairwise correlations between these measures. We found support for positive correlations (against no correlation or the null) for all but SNI-complexity and SNS-Online, for which the evidence was anecdotal. The strongest correlation was between the two variables derived from the SNI questionnaire (size and complexity). Interestingly, SNI-size was numerically more strongly related to SNI-complexity (r = .64) than it was to SNS-RealWorld (r = .37), and SNS-RealWorld had very similar correlations with the two SNI variables (SNI-Size, r = .37; SNI-complexity, r = .36). These results in general support the validity of this group of measures as tapping into a common construct, with SNS-Online being more different from the other measures.

**Table 1:** Means and descriptive statistics for the SNS measures

	Mean (SD)	Min	Max	Skewness	Kurtosis	Reliability (GLB)
SNI-Size	5.71 (1.84)	2.65	10.86	0.75	0.32	.65
SNI-complexity	3.49 (1.44)	1.00	8.00	0.21	0.17	.60
SNS-Online	23.02 (13.29)	0.00	64.81	0.31	0.33	NA
SNS-RealWorld	0 (0.63)	-1.56	1.85	0.50	0.18	.88

Reliability was estimated using the greatest lower bound (GLB), which is conservative and does not require assumptions about the equivalence of item variances or covariances (Jackson & Agunwamba, 1977). SNI-Size was transformed using a square-root. SNS-Real world items were transformed using a square root and z-scored before they were averaged

**Table 2:** Correlations between SNS measures with Bayesian support

	Pearson's r	95% CI	BF_+0_
SNI-Size - SNI-complexity	.64	.51 , .73	2.16E+12 **
SNI-Size - SNS-Online	.22	.05 , .38	3.714 *
SNI-Size - SNS-RealWorld	.37	.19 , .51	761.952 **
SNI-complexity - SNS-Online	.19	.03 , .36	1.77
SNI-complexity - SNS-RealWorld	.36	.19 , .50	609.198 **
SNS-Online - SNS-RealWorld	.34	.16 , .48	188.686 **

For all tests, the alternative hypothesis specifies that the correlation is positive. An uninformative stretched beta prior of 1 was used because previous results indicated a wide range of correlations: *moderate and **extreme support for H1 (Lee & Wagenmakers, 2014)

BI2011 included participants with a wide range in age (19–83 years). Likewise, we did not restrict age in the current sample (our sample had an age range of 18–63 years). We found evidence in support of a null correlation between all four measures of social network and gender (*r*s between −.09 and .04, BF_01_ between 5.6 and 8.1). We found evidence in support of a null correlation between SNI-complexity as well as SNS-RealWorld and age (*r*s of −.01 and .05, BF_01_ of 8.6 and 7.4 respectively). The last two measures (SNI-Size and SNS-online) also showed small correlations with age with support for or against a correlation inconclusive (*r*s of −.14 and −.16, BF10 of .36 and .51 respectively).

KA2012 did not report the correlation between their SNS-online (number of friends on Facebook) and a mean SNS-RealWorld measure, but they report that SNS-online correlated moderately (.3 < r < .4) with five of the eight items on the SNS-RealWorld scale. They considered the two measures as tapping distinct but related constructs, which is generally consistent with our results.

### Amygdala GMV

Amygdala GMV was in the range reported in the literature (Majrashi et al., 2022): right amygdala (*M* = 1750 mm^3^, *SD* = 175, range = 1139–2191 mm^3^) and left amygdala (*M* = 1773 mm^3^, *SD* = 202, range = 1292–2445 mm^3^). The nuclei comprising the BLA represented 77% of the total amygdala volume, bilaterally. The Ce nucleus comprised only 2% of the total amygdala volume. See Table S1 for volumetric measurements for segmented amygdala nuclei, as well as correlations with eTIV, age, and gender.

Men had significantly larger brains than women (eTIV; *r* = −.61, BF_10_ = 3.902e+10). This also was true for individual ROIs (Table 3 and Supplemental section, including complete volumetric measurements separated by gender). For all further analyses, we will consider amygdala total and subregion volumes controlling for eTIV, age, and gender (Table 4).

**Table 3:** Volumetric measurements (mm^3^) and correlation values (r), by hemisphere

Mean (SD)	Min	Max	Skewness	Kurtosis	Correlation with age	Correlation with gender
All participants (117)						
R Whole Amygdala 1753 (169)	1349	2191	0.10	2.90	**.02 (0.12)**	**-.31 (35.43)**
L Whole Amygdala 1775 (202)	1340	2445	0.40	3.20	**.05 (0.14)**	**-.38 (876.66)**
R BLA 1351 (131)	1034	1671	0.10	2.90	**.02 (0.12)**	**-.30 (21.60)**
L BLA 1368 (156)	1040	1883	0.30	3.30	**.05 (0.13)**	**-.37 (572.86)**
R Ce 41 (7)	29	57	0.20	2.50	**.06 (0.15)**	-.20 (1.29)
L Ce 40 (8)	20	62	0.30	3.80	**.13 (0.31)**	**-.27 (7.38)**
R Hippocampus 4038 (305)	3208	4855	0.30	3.00	**.06 (0.14)**	**-.28 (12.62)**
L Hippocampus 3879 (355)	2920	4791	0.00	3.20	.15 (0.41)	**-.13 (0.30)**

For the correlations, males were coded as 0. A negative correlation with gender indicates a larger volume in men compared to women. Results are reported as Pearson correlations with BF10 in parentheses, using an uninformative stretched beta prior of 1. Values in bold have BFs >3 or <.33

**Table 4:** Correlations between SNI-Size and SNI-Complexity and the size of different structures

	N	L Amy.	R Amy.	L Hipp.	R Hipp.	L BLA	R BLA	L Ce	R Ce
Bickart (2011)									
SNI-Size	58	**.38 (.01)**	**.29 (.04)**	.23 (.10)	.10 (.47)	-	-	-	-
SNI-Complexity	58	**.39 (.00)**	**.30 (.02)**	.25 (.06)	.15 (.29)	-	-	-	-
Our study									
SNI-Size	117	.09 (0.55)	.14 (1.25)	.14 (1.20)	.12 (0.88)	.09 (0.59)	.13 (1.10)	.06 (0.42)	*-0.05 (0.16)*
SNI-Complexity	117	.11 (0.80)	.10 (0.64)	*.01 (.25)*	.08 (0.53)	.11 (0.81)	.10 (0.62)	.05 (0.34)	*-.09 (0.12)*

Bickart’s results are standardized regression coefficients and *p*-values in parentheses. Our results are reported as Pearson correlations with BF+0 in parentheses, using an informative stretched beta prior of .3, which assumes that 80% of the correlations are likely to be between 0 and .5. Values in bold are considered significant or are supported by a BF+0 >3. Values in italics have a BF+0 <.33, in support of a null or negative correlation. Some BF01 are also reported in the text to test support for the null

### Replication of BI2011

BI2011 reported that volume of the right and of the left amygdala was positively correlated with both SNI-Size and SNI-Complexity. Their sample was 58 adults (22 women; age range 19–83 years). They reported correlations by age group and by gender and generally found the same results in all groups with some differences in magnitude (e.g., larger correlations in younger than older participants, larger correlation for SNI-Size in women than men). However, their sample size was smaller and neither age nor gender was tested as a moderator. The authors included the left and right hippocampus as control regions, which we also do here (Jones et al., 2020a, 2020b). Our partial correlations control for total brain volume, gender, and age. Aside from these confirmatory analyses, we also include exploratory correlations with nuclei of the amygdala (BLA and Ce).

**Fig. 1:**
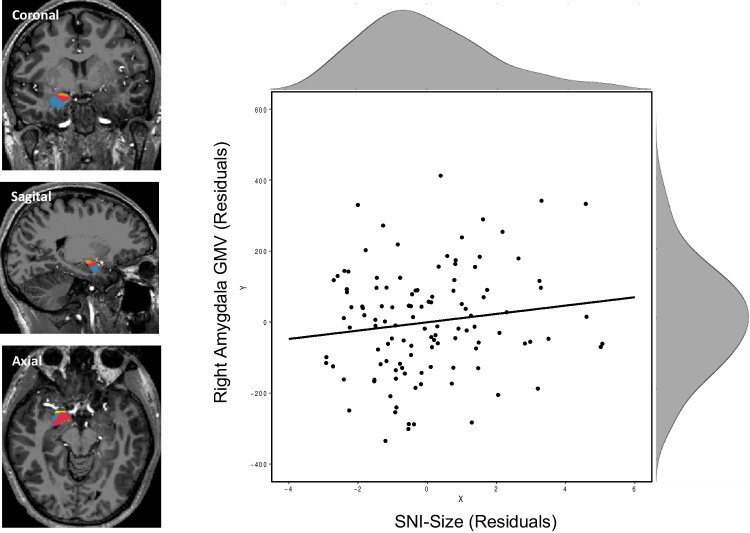
Left: One representative subject with right amygdala segmentation overlaid, shown in three views. Separate colors represent segmented nuclei, whose GMVs were summed to form the R Whole Amygdala measurement. Right: Scatterplot showing the relation between SNI-Size and R Whole Amygdala, when controlling for total brain volume, gender, and age. Histograms illustrate the distribution of scores along each dimension

Our results do not qualitatively replicate the conclusions of BI2011, as we do not find a positive correlation for the Amygdala with social network size or complexity measures. All BF_+0_ are between .33 and 1, revealing anecdotal support for our H_0_ that the correlation is either null or negative. Figure 1 plots the strongest correlation between SNI-Size and right amygdala volume. When considering the effect of priors, we note that the BF_+0_ never reached 3 for any prior (the maximum was 2.23, at a stretched beta prior of .03). The BF_01,_ providing a test comparing the support for the null relative to support for a correlation also were inconclusive (all < 3).

The exploratory analyses in the nuclei of the amygdala were generally consistent with these results. In some cases, we find moderate support for the null (SNI-Size with L Ce: BF_01_ = 3.58; SNI-Size with R Ce: BF_01_ = 3.96; SNI-Complexity with L Ce: BF_01_ = 3.980).

When considering correlations of SNI-Size with hippocampus volume, we likewise find that most correlations show only anecdotal support for H_0_, as the correlation is either null or negative but small. The BF_01,_ which provide a test comparing support for the null relative to support for a correlation, were also inconclusive (all < 3). However, in the case of the correlations between SNI-Complexity with the volume of the hippocampus, we find moderate support for the null (R Hipp: BF_01_ = 3.03; L Hipp: BF_01_ = 4.41).

An important question focuses on what could be reasonably expected in any such specific replication attempt. To facilitate this discussion, Spence & Stanley (2016) advocate the use of a prediction interval (PI). The PI captures both the uncertainty in the population parameter and the inherent variability in individual observations and, as such, it tends to be larger than the confidence interval. For instance, consider again the largest correlation we observed. Given the initial effect size observed (relating the volume of the right Amygdala and SNI-size), the sample size of the initial study (58) and that of the current study (117), the PI is 95% PI [−.01, .57]. This PI captures 95% of the replication results solely because of sampling error and acknowledging that the true effect size estimated by the first study is unknown. The PI depends on both the size of the initial study and of the replication. Of note, even a very large initial study sample size (e.g., 2000) would still yield a 95% PI that includes the observed correlation [.13, .46]. Likewise, if we had collected data from 2000 participants and observed an effect size of r = .14 (which clearly would have been significant in that case), it also would fall within the 95% PI [.03, .51]. The PI is highly sensitive to the effect size; for instance, the 95% PI for our replication of the correlation between left amygdala and SNI-Size is [.10, .64], and our observed value of r = .09 falls outside of it. The reader is encouraged to explore the space of possibilities with the online calculator published by (Spence & Stanley, 2016): https://replication.shinyapps.io/correlation/. What this illustrates is that single replication studies (indeed, single studies) are very limited and, because sampling error is unavoidable, especially for small to medium effect sizes, a meta-analytical mindset is the best approach (Margoni & Shepperd, 2020).

### Analysis based on KA2012

**Fig. 2:**
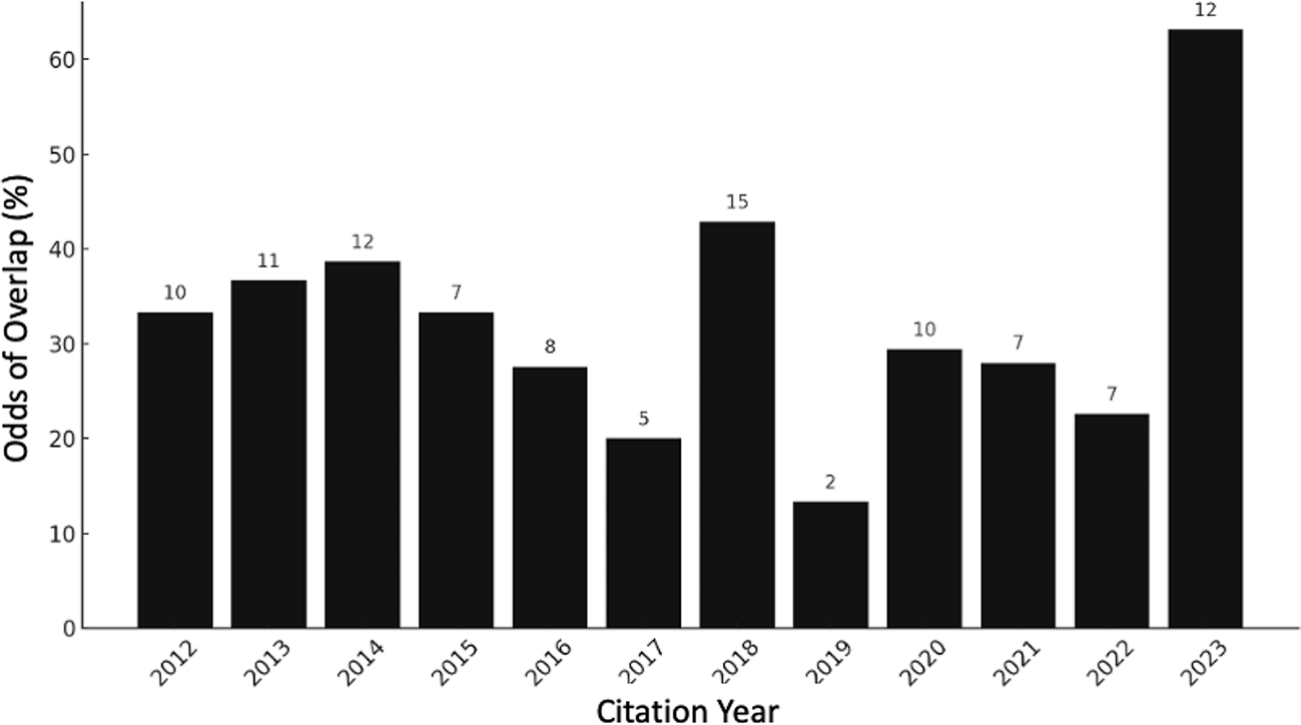
Odds of overlap between BI2011 and KA2012. Above each bar is the number of citations. Citations from Web of Science, October 15, 2023

KA2012 reported that GMD in the amygdala was correlated with both the size of the online social network (Facebook number) and real-world social network. We performed a co-citation analysis of BI2011 and KA2012, extracting all the citations of both articles from Web of Science (as of October 2023), and measuring the percentage of articles citing BI2011 that also cite KA2012. The results (Fig. 2) revealed that since BI2011 was published, 32% of the times it is cited, KA2012 also is cited. As of October 15, 2023, this rate has grown to 63.2%. Looking at the 12 papers cited in the past year, five articles (Driver et al., 2023; Dunbar & Shultz, 2023; Monninger et al., 2023; Tusche et al., 2023; Vandenbulcke et al., 2023) cited the two studies together or in contiguous sentences, and explicitly (incorrectly) referred to them as both using GMV. Another three articles (Goldman, 2023; Lu et al., 2023; Noonan et al., 2023) cited both without mentioning a dependent variable, whereas only four articles (Duffner et al., 2023; Fulford & Holt, 2023; Kieckhaefer et al., 2023; Rollings et al., 2023) suggested there was a difference in dependent variable (although the specifics were sometimes inaccurate, with the wrong measure attributed to one or both studies). This situation may not be surprising; after years of systematic co-citation, authors increasingly rely on gist knowledge and secondary sources. As a result, KA2012 is increasingly likely to be seen as replicating the link between amygdala GMV and the size of real-world social network, as well as extending this result to the size of an online social network. However, it never even reported GMV.

Other factors complicate the interpretation of KA2012**.** This article reports on four experiments. The first (n = 125) related GMM to SNS-Online in an exploratory manner over the entire brain but also in the amygdala specifically using an ROI analysis, motivated by the BI2011 result. The second experiment was a replication (n = 40), considering only the amygdala and three other ROIs (left MTG, right STS, and right entorhinal cortex) that showed evidence for a correlation in their first experiment. The third experiment was behavioral only, looking at the relation between SNS-Online and SNS-RealWorld. The fourth experiment (n = 65) was a reanalysis of GMM data from participants already included in Experiments 1 and 2, but who provided new survey data in Experiment 3, such that correlations of GMM could be evaluated relative to both SNS-Online and SNS-RealWorld.

Given this, it is unclear what should be considered the best estimates of the correlations with SNS-Online and amygdala volume (their stated question of interest), because the two estimates they provide are not independent (from E1: n = 125, r = .322 in the right AMY and r = .298 in the left AMY; from E2: n = 65, r = .182 in the right AMY and r = .135 in the left AMY). The only estimates of correlations with SNS-RealWorld come from E2 (n = 65, r = .264 in the Right AMY and r = .192 in the Left AMY). We chose to use the E1 estimates for SNS-Online, because it is the largest sample, which can provide the most precise estimate, and the only estimate for SNS-RealWorld comes from E2 with a smaller sample.

**Fig. 3:**
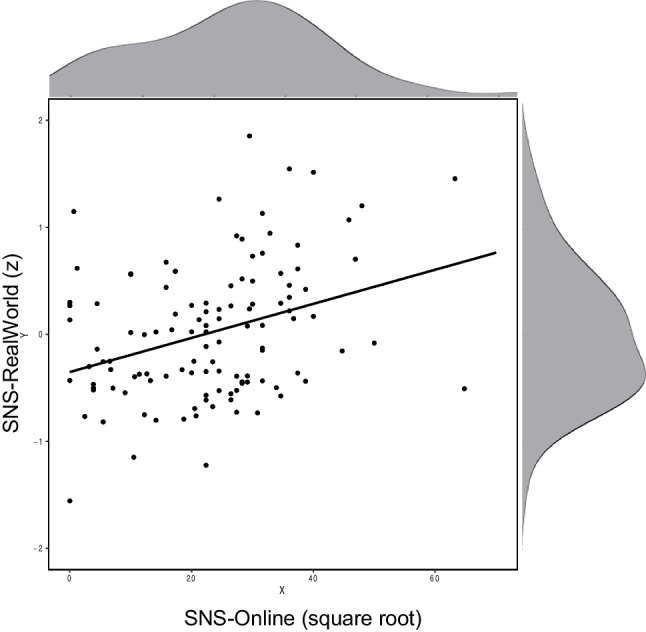
Relationship between SNS-Online and SNS-RealWorld. Histograms illustrate the distribution of scores along each dimension

KA2012’s E3 examined the relationship between SNS-Online and SNS-RealWorld, but they surprisingly did not report the exact correlation between the two indices. They do report that SNS-Online is larger than SNS-RealWorld and that most of the items on the SNS-RealWorld questionnaire correlate positively with SNS-Online. In the present dataset, the correlation between SNS-RealWorld and SNS-Online was r = .34 (Table 2, Fig. 3). Although they noted that the two variables are related, KA2012 also conducted analyses (based on E2, n = 65) on GMV to compute the unique correlation with SNS-Online, controlling for SNS-RealWorld, and vice versa.

Table 5 reports our results of GMV for SNS measures. This analysis is not a replication of KA2012, but instead is an attempt to test what many have taken to be the conclusions of KA2012 as a replication of BI2011.

**Table 5:** Results of GMV for SNS measures

	N	L Amy.	R Amy.	L Hipp.	R Hipp.	L BLA	R BLA	L Ce	R Ce
KANAI (2012)									
DV is GMM									
SNS-Online	125	**.30 (.003)**	**.32 (.001)**	-	-	-	-	-	-
SNS-RealWorld	65	.19 (.13)	**.26 (<.05)**	-	-	-	-	-	-
SNS-Online unique	65	.09 (.48)	0.11 (.41)	-	-	-	-	-	-
SNS-RealWorld unique	65	not reported (p>.05)	**.29 (P<.05)**	-	-	-	-	-	-
Our study									
DV is GMV									
SNS-Online	117	*-.03 (0.18)*	*-.08 (0.13)*	*-.04 (0.17)*	*-.10 (0.12)*	*-.02 (0.19)*	*-.10 (0.12)*	*.02 (0.28)*	*.08 (0.52)*
SNS-RealWorld	117	.16 (1.83)	*.04 (0.31)*	*.02 (0.26)*	*.00 (0.22)*	*.14 (1.3)*	*.03 (0.281)*	**.21 (4.62)**	*.02 (0.26)*
SNS-Online unique	117	*-.09 (0.12)*	*-.09 (0.12)*	*-.05 (0.16)*	*-.10 (0.12)*	*-.08 (0.13)*	*-.11 (0.11)*	*-.01 (0.16)*	.08 (0.52)
SNS-RealWorld unique	117	.18 (2.78)	.07 (0.43)	*.03 (0.30)*	*.03 (0.30)*	.16 (1.79)	.06 (0.42)	**.21 (5.10)**	*-.01 (0.20)*

Kanai’s results are Pearson *r*s, and *p*-values as reported in the article. Our results are reported as Pearson correlations with BF+0 in parentheses, using an informative stretched beta prior of .3, which assumes that 80% of the correlations are likely to be between 0 and .5. Values in bold are considered significant or are supported by a BF+0 >3. Values in italics have a BF+0 <.33, in support of a null or negative correlation. Some BF01 are also reported in the text to test support for the null

Our results do not provide support for the claim that the GMV of the amygdala correlates with either SNS-Online or SNS-RealWorld. In fact, considering SNS-Online for bilateral amygdalae and SNS-RealWorld for the right amygdala, we found the BF_+0_ was < .33, offering support against the presence of a positive correlation. In all three cases when we directly compared support for the presence versus the absence of a correlation, we found moderate support for a null correlation (BF_01_ > 3).

**Fig. 4:**
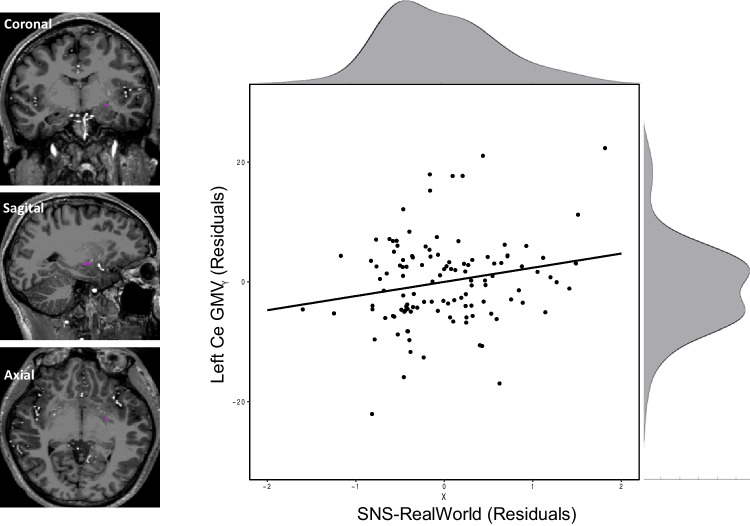
Left: One representative subject with Left Ce Nucleus overlaid in magenta, shown in three views. Right: Relationship between SNS-RealWorld and Left Ce grey matter volume, when controlling for total brain volume, gender, and age. Histograms illustrate the distribution of scores along each dimension

The only region where any support was found for a correlation was the Left Ce, with moderate support for a positive correlation between SNS-RealWorld and GMV (r = .21, BF_+0_ = 4.62). This correlation was specific to SNS-RealWorld, as it survived controlling for SNS-Online (r = .21, BF_+0_ = 5.10; Fig. 4).

## Discussion

The initial motivation for this study was to provide a replication of the results in BI2011, which show that amygdala GMV is related to both SNI-Size and SNI-Complexity. Our replication was inconclusive in this regard, with small effect sizes below r = .2 and only anecdotal support for the existence of a positive correlation (but also no conclusive evidence in favor of the null). Only in some cases (the left and right Ce for SNI-Size, the left and right hippocampus, and the left Ce for SNI-Complexity) did we find moderate support for the absence of a correlation between social network measures and GMV.

When an initial study finding positive evidence is followed by another failing to find evidence for the same relationship, authors typically conclude that an effect did not replicate. Based on a discussion of prediction intervals (PIs; Spence & Stanley, 2016), we propose an alternative interpretation. Small effect sizes, such as those initially reported by BI2011, lead to very large PIs. This would be true even if the initial study, or this study, had used considerably larger samples. For instance, even if we had collected a sample of 2000 participants, our observed correlation of r = .14 (SNI-size and right amygdala) would still be included in a 95% PI that assumes the initial result of r = .29 by BI2011 is true, providing only for sampling error. In other words, there is a sense in which it was almost impossible not to replicate the initial result. PIs offer a range within which future observations are expected to fall, given the data and model from a prior study. If the result of a new study falls within this interval, it is tempting to consider the study as a successful replication. In one sense, however, the large PI reveals the futility of trying to conduct a replication of such a small correlation. Spence & Stanley (2016) argue that little useful knowledge can be derived from any single study and that we should instead shift to a meta-analytic approach to estimating effect sizes in which we are interested.

One challenge of a meta-analytic approach is that of pooling together results that measure effects between the same construct. A multiplicity of different measures for the same construct can be a good thing. It can help strengthen claims when the different measures function as convergent indicators of the same construct. Analyses that use correlated indicators to define constructs as latent variables (Bollen, 2002) can support analytical methods that allow use to investigate relationships that focus on construct-relevant variance, supporting stronger inferences (Engle et al., 1999; Richler et al., 2019). However, when different measures are considered to be related to the same construct and they do not actually intercorrelate, progress about understanding the relationships of the target construct to other variables is limited (Gauthier, 2020; Rezlescu et al., 2017).

With regards to evaluating whether the initial BI2011 result has been replicated in the literature, our work provides good support that the four different measures of social network used in BI2011 and KA2012 are to some degree influenced by a common factor. Experimental results originating from these different indices of social network may be meaningfully combined and compared (e.g., ideally in a latent variable framework). In contrast, there are important reasons to be careful about the commensurability of GMV to other measures that have been summarized as also indexing “volume,” in particular GMM, as used in KA2012. KA2012 is very often (and increasingly over the years) cited as providing a replication of BI2011. Because we did not perform VBM on our data, we cannot provide evidence for the correlation between GMV and GMM, but other work suggests they may yield quite distinct influences (Gennatas et al., 2017a, 2017b; Goto et al., 2022a, 2022b).

Following up on the results of KA2012, we provided a “replication” of a different nature—not one for the result they actually reported on correlations with GMM, but one that addresses the implied replication and extension of the GMV result that BI2011 reported. In other words, if KA2012’s results were indeed on GMV, as they have often been incorrectly cited for, would we replicate them here? We did not obtain support for the positive correlations reported in KA2012, and in several cases obtained moderate support for a null correlation. Even by the PI approach, our results often fall outside the 95% PI for this replication (e.g., the 95% PI for the correlation of r = .32 between right amygdala and SNS-Online would be [.10, .54], and we observed a correlation of r = −.08). Therefore, in addition to the principled reasons for why KA2012 does not represent a replication of BI2011 (i.e., they use different morphometric measures), these results provide some conclusive evidence that the effect does not in fact replicate.

In the present work, the only support for a positive correlation was obtained in the left Ce nucleus of the amygdala, with SNS-RealWorld. We did not expect this specific relationship, and we note that it was not found when using different measures of the size of real-world social network (those based on the SNI; Table 5). The 95% PI for a future large replication of this effect (n = 1000) is [.03, .38]. One recent project considering amygdala nuclei volume observed a similar pattern in the Ce nucleus, reporting that individuals with larger central nucleus volumes had larger social networks (Jones et al., 2020a, 2020b). We caution the reader when interpreting these results, however, given that (1) Jones et al. (2020a, 2020b) only considered homeless and precariously housed individuals, (2) social network size was assessed via a structured interview design, and (3) Jones’ secondary analysis with the same data showed no significant association between Ce nucleus and network size.

## Conclusions

With a sample size of 128 subjects scanned at high resolution, we examined the relationship between social network size and the grey matter volume of the amygdala and its nuclei. Our results highlight the relevance of matching critical morphometric measurement tools before directly comparing results across studies. We conclude that two often co-cited projects—KA2012 and BI2011—are in fact not comparable because of their unique morphometric measurements. Our results highlight the value of a focused and narrow lens in evaluating evidence for replication over the years.

### Supplementary Information

Below is the link to the electronic supplementary material.

Supplementary file1 (DOCX 1402 KB)

## Data Availability

The derived summary data (at the level of individual participants) are publicly available at FIGSHARE at https://doi.org/10.6084/m9.figshare.24714897.v1.

## Footnotes

1 This is to account for volume changes between the individual’s native space image and a registration template.

## Declarations

### Open practices statement

The experiment was not preregistered and data are available at FIGSHARE at https://doi.org/10.6084/m9.figshare.24714897.v1.

### Author Note

This research was supported by NSF BCS award 1840896 to I.G.
