# Supplementary material for: Challenges in replication: Does amygdala gray matter volume relate to social network size?
Source: Cogn Affect Behav Neurosci. 2024 Mar 28;24(4):707–19. doi: 10.3758/s13415-024-01185-w (PMC11233388; doi:10.3758/s13415-024-01185-w)
Supplement: Supplementary file 1 — Supplementary file1 (DOCX 1402 KB) [file 13415_2024_1185_MOESM1_ESM.docx]

Supplement

Social Network Size questionnaires :

**Kanai et al. (2012) Social Network Size Questionnaire**

1. How many were present at your 18th or 21st birthday party?
2. If you were going to have a party now, how many people would you invite?
3. What is the total number of friends in your phonebook?
4. Write down the names of the people to whom you would send a text message marking a celebratory event (e.g. Birthday, Christmas, new job, good exam result, etc.). How many people is that?
5. Write down the names of people in your phonebook you would meet for a chat in a small group (one to three people). How many people is that?
6. How many friends have you kept from school and university whom you could have a friendly conversation with now?
7. **How many friends do you have on ‘Facebook’?*
8. How many friends do you have from outside school or university?
9. Write down the names of the people of whom you feel you could ask a favor and expect to have it granted. How many people is that?

***We replaced Question #7 with the following questions:**

1. How many different social media platforms (e.g., Facebook, Twitter, Instagram etc.) do you use on a regular basis (at least once a week?)
2. How many friends or mutual followers (people you follow who ALSO follow you) do you have across all platforms? (give your best estimate)
3. Some individuals may be friends or mutual followers with you on several platforms. How many UNIQUE friends or mutual followers do you have total? A person only counts once, even if you encounter them on many platforms. Give your best estimate.

**Cohen et al. (1997) Social Network Index**

Instructions: This questionnaire is concerned with how many people you see or talk to on a regular basis including family, friends, workmates, neighbors, etc. Please read and answer each question carefully. Answer follow-up questions where appropriate.

1. Which of the following best describes your marital status?
   - (1) currently married & living together, or living with someone in marital-like relationship
   - (2) never married & never lived with someone in a marital-like relationship
   - (3) separated
   - (4) divorced or formerly lived with someone in a marital-like relationship
   - (5) widowed
2. How many children do you have? (If you don't have any children, check '0' and skip to question 3.)
   - 0
   - 1
   - 2
   - 3
   - 4
   - 5
   - 6
   - 7 or more

2a. How many of your children do you see or talk to on the phone at least once every 2

weeks?

- - 0
  - 1
  - 2
  - 3
  - 4
  - 5
  - 6
  - 7 or more

1. Are either of your parents living? (If neither is living, check '0' and skip to question 4.)
   - (0) neither
   - (1) mother only
   - (2) father only
   - (3) both

3a. Do you see or talk on the phone to either of your parents at least once every 2 weeks?

- - (0) neither
  - (1) mother only
  - (2) father only
  - (3) both

1. Are either of your in-laws (or partner's parents) living? (If you have none, check the appropriate space and skip to question 5.)
   - (0) neither
   - (1) mother only
   - (2) father only
   - (3) both
   - (4) not applicable

4a. Do you see or talk on the phone to either of your partner's parents at least once every 2

weeks?

- - (0) neither
  - (1) mother only
  - (2) father only
  - (3) both

1. How many other relatives (other than your spouse, parents & children) do you feel close to? (If '0', check that space and skip to question 6.)
   - 0
   - 1
   - 2
   - 3
   - 4
   - 5
   - 6
   - 7 or more

5a. How many of these relatives do you see or talk to on the phone at least once every 2

weeks?

- - 0
  - 1
  - 2
  - 3
  - 4
  - 5
  - 6
  - 7 or more

1. How many close friends do you have? (meaning people that you feel at ease with, can talk to about private matters, and can call on for help)
   - 0
   - 1
   - 2
   - 3
   - 4
   - 5
   - 6
   - 7 or more

6a. How many of these friends do you see or talk to at least once every 2 weeks?

- - 0
  - 1
  - 2
  - 3
  - 4
  - 5
  - 6
  - 7 or more

1. Do you belong to a church, temple, or other religious group? (If not, check 'no' and skip to question 8.)
   - no
   - yes

7a. How many members of your church or religious group do you talk to at least once every 2 weeks? (This includes at group meetings and services.)

- - 0
  - 1
  - 2
  - 3
  - 4
  - 5
  - 6
  - 7 or more

1. Do you attend any classes (school, university, technical training, or adult education) on a regular basis? (If not, check 'no' and skip to question 9.)
   - no
   - yes

8a. How many fellow students or teachers do you talk to at least once every 2 weeks? (This includes at class meetings.)

- - 0
  - 1
  - 2
  - 3
  - 4
  - 5
  - 6
  - 7 or more

1. Are you currently employed either full or part-time? (If not, check 'no' and skip to question 10.)
   - no
   - yes, self-employed
   - yes, employed by others

9a. How many people do you supervise?

- - 0
  - 1
  - 2
  - 3
  - 4
  - 5
  - 6
  - 7 or more

9b. How many people at work (other than those you supervise) do you talk to at least once every 2 weeks?

- - 0
  - 1
  - 2
  - 3
  - 4
  - 5
  - 6
  - 7 or more

1. How many of your neighbors do you visit or talk to at least once every 2 weeks?
   - 0
   - 1
   - 2
   - 3
   - 4
   - 5
   - 6
   - 7 or more
2. Are you currently involved in regular volunteer work? (If not, check 'no' and skip to question 12.)
   - no
   - yes

11a. How many people involved in this volunteer work do you talk to about volunteering-

related issues at least once every 2 weeks?

- - 0
  - 1
  - 2
  - 3
  - 4
  - 5
  - 6
  - 7 or more

1. Do you belong to any groups in which you talk to one or more members of the group about group- related issues at least once every 2 weeks? Examples include social clubs, recreational groups, trade unions, commercial groups, professional organizations, groups concerned with children like the PTA or Boy Scouts, groups concerned with community service, etc. (If you don't belong to any such groups, check 'no' and skip the section below.)
   - no
   - yes

12a. Consider those groups in which you talk to a fellow group member at least once every

2 weeks. Please provide the following information for each such group: the name or

type of group and the total number of members in that group that you talk to at least

once every 2 weeks.

| Group | Total number of group members that you talk to at least once every 2 weeks |
| --- | --- |
| 1. |  |
| 2. |  |
| 3. |  |
| 4. |  |
| 5. |  |
| 6. |  |

Table S1. Volumetric measurements (mm^3^) and correlation values (r) for segmented amygdala nuclei, separated by hemisphere.

Table S2. Volumetric measurements (mm^3^), by gender and hemisphere
